# Effects of Obesity on Infections with Emphasis on Skin Infections and Wound Healing

**DOI:** 10.29245/2767-5092/2022/3.1157

**Published:** 2022

**Authors:** Daniela Frasca, Natasa Strbo

**Affiliations:** 1 Department of Microbiology and Immunology, University of Miami Miller School of Medicine, Miami, FL USA; 2 Sylvester Comprehensive Cancer Center, University of Miami Miller School of Medicine, Miami, FL USA

**Keywords:** Obesity, Infections, Wound healing

## Abstract

Obesity represents a serious health problem as it is rapidly increasing worldwide. Obesity is associated with reduced health span and life span, decreased responses to infections and vaccination and increased frequency of inflammatory conditions. In this review, we summarize published data showing that obesity increases the risk of different types of infections, with a special focus on skin infections. Obesity also induces skin changes and conditions (inflammation-based and hypertrophic) which are often associated with fungi or bacteria overgrowth. The association of obesity with the skin microbiome has been established in both mice and humans. Balance of commensal microbes controls skin homeostasis and the host immune response, while changes in normal physiologic skin microbiome composition and pathologic bacteria contribute to skin diseases. We also summarize the major steps in wound healing and how obesity affects each of them. The role that immune cells have in this process is also described. Although the studies summarized in this review clearly demonstrate the deleterious effects of obesity on wound healing, additional studies are needed to better characterize the cellular and molecular mechanisms involved and identify specific targets of intervention.

## Introduction

Obesity is a complex inflammatory chronic condition that affects both children and adults and has become a worldwide epidemic. Diets enriched in fat and calories and a sedentary lifestyle with limited physical activity are usually blamed for the increase in the prevalence of obesity. The most visible sign of obesity is accumulation of body fat ^1^, usually measured by body mass index (BMI) ≥ 30 kg/m^2^ as stated by the Centers for Disease Control and Prevention (CDC). BMI however only measures total body weight without taking into account changes in body fat ^2^ as well as the location of the fat, visceral versus subcutaneous, which is important as the accumulation of fat between different depots is more important than the amount of total fat in the body for the risk of developing obesity-associated conditions. The subcutaneous adipose tissue (AT) is generally located in lower parts of the body, and is measured by hip, thigh and leg circumference, but can also accumulate in the area around the neck. The visceral AT surrounds internal organs and includes omental, mesenteric, epididymal, perirenal, retroperitoneal, epicardial ^3^. The subcutaneous accounts for almost 80% of human AT, but the visceral is more metabolically active and inflammatory, and its accumulation is a greater predictor of obesity-associated mortality ^4^.

As indicated by the CDC (https://www.cdc.gov/healthyweight/effects/index.html), obesity decreases healthspan and lifespan and increases premature mortality leading to significant rise in individual, national and global healthcare costs. Obesity increases the risk to develop debilitating diseases such as cardiovascular disease, type-2 diabetes mellitus, cancer, atherosclerosis, Alzheimer’s disease and dementia, inflammatory bowel disease ^5,6^. Obesity also increases the risk of health conditions like musculoskeletal disorders and chronic back/lower limb pain, infertility and gestational problems, respiratory problems, and is a well-known risk factor for insulin resistance. Importantly, obesity-driven chronic diseases can establish a vicious cycle of inflammation and damage, leading to persistent dysfunctional immunity ^7–9^.

## Obesity Increases the Risk of Infections

Obesity influences not only the risk of getting various infections but also the outcome of the infection. There is a large amount of published work showing the effects of obesity on respiratory tract infections (RTIs), with obese individuals being at higher risk to contract both bacterial and viral infections as compared to lean controls ^10,11^. Mechanistically, lung function has been shown to be altered in individuals with obesity ^12^, with increased airway resistance causing an increase in work of breathing and respiratory rates due to the increased fatigue needed to inflate the lungs ^12^. Individuals with obesity also experience higher weight load on the thorax, which is independent of any underlying parenchymal lung disease. Moreover, cells in the lung have been shown to be able to secrete leptin ^13,14^, the adipokine primarily made by the AT, with effects on both systemic and pulmonary inflammation through secretion of leukotrienes by alveolar macrophages ^15^. A strong association has been shown between obesity and severity of illness after infection with the A/H1N1pdm09 influenza virus ^16^ or with the coronavirus SARS-CoV-2, cause of the COVID-19 pandemic ^17^. Obesity affects both quantity and quality of the antibody responses in COVID-19 patients. We found that SARS-CoV-2 IgG antibodies are negatively associated with BMI, as expected based on the known effects of obesity on humoral immunity ^18^. Moreover, when we evaluated the quality of the antibody response in lean and obese COVID-19 patients, as compared to uninfected controls without previous history of autoimmunity, we found that the sera of adult COVID-19 patients contain less neutralizing antibodies and more antibodies with autoimmune specificities ^19^. Due to immunosenescence, older adults with obesity are in general at higher risk of overall infection, as compared to younger controls, as shown by increased mortality associated with influenza ^20^.

In addition to RTIs, obesity has been associated with gastric infections with *Helicobacter pilori* ^21^, urinary tract infections ^22^, periodontitis ^23^, post-trauma infections ^24^, post-surgical infections ^25^, viral hepatitis ^26^ and skin infections as detailed below.

The mechanisms by which obesity induces higher susceptibility to infections are not completely known, but several hypotheses have been proposed. First of all, obesity alters the integrity of lymphoid tissues and induces a dysfunctional coordination of innate and adaptive immune responses due to impaired chemotaxis, altered differentiation and function of immune cells, dysregulated cytokine production and imbalanced cross-talk between immune system and adipose cells ^27^.

Because obese individuals are highly susceptible to infections and have a compromised immune system, vaccines may not provide adequate protection to this population. Obesity induces impaired serum responses to the influenza vaccine in children and adults with obesity ^28,29^, as well as in elderly individuals ^30^. A negative association between obesity and vaccine responses was observed in response to hepatitis B ^31^, tetanus ^32^ and rabies ^33^ vaccines.

## Obesity and Skin Infections

Skin is the largest organ in the human body, accounting for 6–10% of total body weight, and accomplishing multiple functions: regulation of body temperature, moisture retention, vitamin D production, and protection of the inner organs from outside pathogens and toxins ^34^. The interplay of hormones, immune signaling molecules, and growth factors is necessary for the establishment of normal skin physiology and dysbalance of this interplay leads to skin changes and may reflects the inner state of the organism ^35,36^.

A large frequency of individuals with obesity (50%) display skin changes such as mechanical friction, skin hypertrophic conditions (acanthosis nigricans, and fibromas or skin tags) and skin infections ^37^. Staphylococcal and streptococcal infections of the skin are the most common gram-positive infections in obesity ^38,39^, which usually present with scrotal cellulitis (erysipelas) or atrophic round scars, secondary to the resolution of bacterial folliculitis ^37^.

Intertrigo, an inflammation-based condition caused by skin-to-skin friction, in warm, moist areas of the body (groin, between folds of skin on the abdomen, under the breasts, under the arms or between the toes), is very often associated with obesity, and is most commonly associated with Candida or Gram-positive bacteria overgrowth ^37,40^. Another skin fold infection observed in obesity is erythrasma, a corynebacterial skin fold infection ^37,41^. Fungal infection of the nails that causes discoloration, thickening, and separation from the nail, onychomycosis, is also commonly observed in obese individuals. Overweight and obese patients with this condition are therapy resistant ^37,42^.

The composition of the skin microbiota is variable and primarily depends on physiology of the skin sites which is affected by dryness, moisture, the amount of sebum, and temperature ^43^. Early microbiome studies established the link between obesity and the human gut microbiome ^44^. More recently, the association between obesity and skin microbiome was established in mice and humans ^45^. Alpha diversity, beta diversity and community composition was significantly different between underweight, normal weight and overweight/obese individuals, characterized by an overpopulation of genera enriched in overweight/obese individuals, including *Anaerococcus, Finegoldia and Peptoniphilus* ^46^. Additionally, it was shown that *Corynebacterium* relative abundance is significantly correlated with BMI, suggesting that it may be used as a marker for obesity and potentially other manifestations of the metabolic syndrome. Moreover, skin commensals, including *Staphylococcus epidermidis*, *Staphylococcus hominis* and *Propionibacterium acnes*, are significantly diminished in another skin condition, hidradenitis suppurativa (HS) skinfolds, while pathogenic *Staphylococcus aureus* dominates at the advanced stages of the disease ^47^. The balance of commensal microbiomes affects skin homeostasis and a host immune response, and dysbiosis or a change in normal physiologic skin microbiome and pathologic bacteria can contribute to skin diseases such as HS. Dysbiosis in obesity should therefore be studied in more details.

## Obesity and Wound Healing

The process of wound healing begins shortly after trauma or inury and involves resident cells (dermal fibroblasts), immune cells recruited to the site of injury (monocytes, macrophages and neutrophils), extracellular matrix (ECM) proteins and growth factors ^48^. Wound healing is a highly coordinated complex process consisting of four main steps: hemostasis, inflammation, proliferation, remodeling. It starts with the formation of a fibrin clot at the injured site, with entrapment of blood cells, platelets and ECM proteins. Then the inflammatory process leads to the secretion of pro-inflammatory cytokines able to recruit inflammatory immune cells and to the removal of dead cells and foreign particles/bacteria. Epithelialization starts at the edge of the wound through the proliferation, migration and differentiation of fibroblasts, keratinocytes and endothelial cells. This process continues until the wound is covered by the thickened mature skin. In the wound healing process, the balance between proliferation, migration, differentiation and apoptosis is critical in the formation of a multilayer functional tissue. Although not many mechanistic experiments have rigorously shown a link between obesity and each step of wound healing, it has been hypothesized that obesity, being an inflammatory condition, may be associated with delayed healing. The reasons are multiple and may be summarized as follows: 1) adipocytes become larger under obese conditions but there is not a concomitant increase in the vasculature and therefore the rate of angiogenesis is delayed as compared to the rate of adipocyte enlargement ^49^. Moreover, larger adipocytes secrete several inhibitors of angiogenesis such as angiostatin and endostatin ^50,51^; 2) the increase in adipocyte size is also associated with areas of hypoxia ^52,53^, due to the insufficient amount of blood vessels needed to oxygenate the tissue, leading to damaged blood capillaries in the wound and higher rates of infections. Moreover, hypoxic wounds impair the synthesis of collagen, leading to defecting healing ^54,55^; 3) vasculature defects are associated with defective/delayed recruitment of immune cells to the wound ^56^, longer inflammatory responses and decreased secretion of mediators; nutritional defects and micro- and macro-nutrients deficiencies in obese individuals also delay the healing process ^57,58^.

The defects in angiogenesis described above, as well as defects in the secretion of angiogenic agents (leptin, angiopoietin, Vascular Endothelia Growth Factor, Transforming Growth Factor-β) by both resident and recruited cells, may lead to chronic wounds due to a slow rate of healing ^59^. The presence of microorganisms in a wound bed can also significantly impair the process of wound healing and lead to stalled, chronic wounds ^60^. It has been hypothesized that microorganisms persist in chronic wounds as a biofilm, refractory to antibiotic and mechanical intervention ^61,62^.

Obesity has been associated with greater risk of surgical site infections ^63^ due to delay in wound healing, which promotes the entrance and proliferation of microorganisms. The breach of the cutaneous barrier during wounding allows microbes (commensal and pathogenic bacteria) from the skin to infiltrate tissues ^64–66^. The presence of bacteria doesn’t immediately indicate a negative implication on wound healing, as there is a spectrum of microbial contamination, colonization, and infection which demonstrates the increasing ability of bacteria to override host immune defenses ^64,65^. However, the chronic wound microenvironment favors bacteria growth due to tissue necrosis, decreased immune response, and low oxygen tension ^47,64^. In conditions of excess weight and pathological expansion of the AT, blood flow is compromised because of the disruption of crosstalk between endothelial cells and adipocytes and production of nitrict oxide is reduced. Nitric oxide is known to be essential for process of vasodilation ^67,68^, hence decreased blood flow causes delayed wound healing. In addition, AT expands without an increase in blood flow (capillary density) and leads to poor perfusion and oxygenation of the AT which further leads to the vascular insufficiency, and also to impaired angiogenesis and chronic inflammation ^52^. Altogether, poor vascularity results in poor oxygenation which further can lead to a delay in normal wound healing.

Another factor that contributes to a poor wound healing in obesity is the immune imbalance characterized by a state of chronic inflammation. Both innate and adaptive immune responses are heavily influenced by AT. The monocytes produce greater amounts of IL-6, IL-12 and TNF-α in response to leptin, the cytokine produced by adipocytes ^69^. Dysfunctional adipocytes in subjects with obesity produce pro-inflammatory cytokines that favor the activation of M1 macrophages (pro-inflammatory) over M2 (anti-inflammatory) macrophages ^70^. Studies *in vitro* have shown that polymorphonuclear neutrophils incubated with leptin produce twice as many reactive oxygen species, as compared to those untreated ^69,71^. Gamma delta (γδ) T cells have been shown to have a crucial effect in wound healing in mouse and human skin ^72^. Murine epidermal γδ T cells are referred to as dendritic epidermal T cells because of the dendritic processes utilized to survey surrounding damaged keratinocytes and secrete large amounts of IFN-γ ^73^, whereas dermal γδ T cells are not dendritic and secrete IL-17 ^74^. Human γδ T cells, conversely, mostly reside in dermis ^75^. In both mice and humans, γδ T cells play a crucial role in the elimination of cutaneous tumors and virally-infected cells ^76^, a function associated with the expression of perforin-2, an innate immune protein responsible for the formation of pores in the membrane of target cells ^77^.

The function of γδ T cells is compromised in obesity, with γδ T cells being unable to regulate keratinocyte homeostasis and with the obese environment further impairing skin structure and cell-to-cell adhesion ^78^.

Other adaptive immune cells that are also affected in obesity include increased inflammatory T helper phenotypes, decreased regulatory T cells, and impaired B cell functions, which inevitably leads to an impaired response against pathogens and therefore causes a higher incidence and more severe disease course in subjects with obesity ^79^.

Adiponectin, also known as adipocyte complement-related protein of 30 kDa (Acrp30), is produced by AT and provides protection against inflammation and oxidative stress. Concentrations of adiponectin are decreased with increasing obesity and adiponectin deficiency affects wound healing in two main ways: first, adiponectin stimulates angiogenesis, so a deficiency of adiponectin means that angiogenesis is impaired; secondly, adiponectin promotes proliferation and migration of keratinocytes, thus a deficiency of adiponectin results in impaired re-epithelialization ^80^.

Collagen synthesis is a necessary part of wound healing and wound integrity. Fibroblasts cannot synthesize collagen properly in an oxygen deficient environment ^81^, so poor oxygenation in AT can predispose obese individuals to delayed wound healing. With the high prevalence of obesity in today’s world and its evidenced effects on health care, there is a need for additional studies to determine the mechanisms underlie impaired wound healing in obesity. The challenge of altering AT’s effect on wound healing should include combination of cytokines, growth factors, nutritional manipulations and surgical techniques.

## Conclusions and Perspectives

Although the studies summarized in this review have indicated the deleterious effects of obesity on wound healing, additional studies are needed to better characterize the cellular and molecular mechanisms involved and identify target-specific therapies. These may include growth factors, cytokines and nutritional supplements that modify adipose tissue’s effects on wound healing.
